# Effectiveness of High Intensity Laser Therapy for Reduction of Pain in Knee Osteoarthritis

**DOI:** 10.1155/2016/9163618

**Published:** 2016-12-20

**Authors:** Anna Angelova, Elena M. Ilieva

**Affiliations:** Medical University of Plovdiv, Plovdiv, Bulgaria

## Abstract

*Introduction*. Osteoarthritis is the most common type of arthritis. It is the main cause of chronic musculoskeletal pain and disability among the elderly population.* Aim*. This is a pilot, randomized clinical study about the effect of high intensity laser therapy in patients with osteoarthritis of the knee (OA of the knee).* Material and Method*. 72 patients (aged between 39 and 83 years) with (clinically and radiographically proved) OA of the knee were included in the study. They were randomized in two groups: therapeutic (test) one (*n* = 37, 65,11 ± 1,40 (mean ± SD) years old; patients were treated with HILT) and control group (*n* = 35, 64,71 ± 1,98; patients receive sham laser). Both groups had seven sessions of treatment. VAS and dolorimetry were used for assessment of pain before and after the therapy. Pedobarometric analysis (static and dynamic) was used to assess comparatively the contact surface area and maximum pressure under the heel.* Results*. Pain levels measured by VAS and dolorimetry decreased significantly in the therapeutic group after seven days of treatment (*p*< 0,001).* Conclusion*. The results after seven days of treatment show more intensive and cumulative effect after the application of high intensity laser therapy in comparison to sham laser. This is the reason why HILT can be a method of choice in the treatment of gonarthrosis.

## 1. Introduction

Osteoarthritis is the most common type of arthritis. It is the main cause of chronic musculoskeletal pain and disability among the elderly population [1, 2]. Osteoarthritis of the knee joint comprises 50% of the rheumatology disorders. Virtually all people over 60 years of age have some degenerative changes in their joints; 70–85% of them OA have signs and symptoms such as pain and short-term morning stiffness [3–5]. Тhe difficulties in daily activities lead to functional limitations, reduced quality of life, and participation in social life restrictions [6]. OA is expected to be the fourth leading cause of disability by the year 2020. According to the last bulletin of WHO, moderate to severe disabilities as a result of osteoarthritis cover 10 mln. оf the population in developed countries and 33.5 mln. of the population in countries with lower income. In general disability as a result of osteoarthritis affects 43.4 mln. of the world population [4, 5, 7]. This defines the medical and social importance of osteoarthritis [6, 7].

Chronic pain, according to many authors, continues for more than six months [2, 8]. Because of that reason continuous therapy is needed [5, 9]. It requires the application of noninvasive treatment methods with proven clinical efficiency. It also stimulates searching of new therapeutic possibilities for reducing the symptoms and increasing functional capacity.

High intensity laser radiation is a relatively new method of application in physical therapy practice, as different mechanisms of action, compared with low intensity laser radiation [10–13]. It is traditionally applied in surgery for the purpose of destruction of tissues. In recent years, some studies were published about the impact of high intensive laser radiation on cell cultures in vitro or on experimental animals. There are a few clinical trials in patients with different disorders [14–19].

*Objective*. The study aims to evaluate the efficiency of high intensity laser therapy in patients with osteoarthritis of the knee.

## 2. Material and Methods

*Including Criteria.* In this study patients with osteoarthritis of the knee (proven clinically) are included, with duration of the symptoms for over 4 years and X-ray stages II and III by Kerllgren and Lawrence; without local application of corticosteroids or hyaluronic acid during the last six months; without physiotherapy during the last six months; being treated with physiotherapy or drugs more than six months before. 

*Excluding Criteria*. Excluding criteria are reactive synovitis (CRP > 6; ESR > 25 mm.) and urine acid above the normal ranges; corticosteroid or hyaluronic acid application during the last six months; malignant tumours; comorbidity contributing to diversions in statics and locomotion or forming contraindications for laser therapy; systematic inflammatory diseases; rejection of the study because of personal reasons.

It is a single blinded, placebo controlled study. According to* the design* of the trial patients are divided in two groups: treatment group and control group. Patients are included in one of the two groups according to the time they come for examination. Every second patient is included in the control group. The patients from the control group are treated with imitation of laser treatment by directing the laser device without turning on the light beam (sham laser). The patients from the treatment group are treated with high intensity laser.

### 2.1. Methods

Semiconductive neodymium laser IV produced by BTL is used, with wave length 1064 nm and maximal power 12 W. The treatment is performed by single application of laser therapy per day, 7 sessions.

The first three procedures are with analgesic effect with dose 12 J/cm^2^ = 300 J for treated area of 25 cm^2^. Laser therapy is applied on the medial and lateral sides of the knee, distant application, for 2 minutes, 25 Hz frequency. The next 4 sessions use biostimulating parameters, applied with dose 120 J/cm^2^ = 3000 J treated area 25 cm^2^, applied on the medial side of the knee, 10 min.

In the study, laser radiation is applied with analgesic parameters on two opposite fields, because, at the selected stages of osteoarthritis of the knee, all intraarticular and periarticular tissues are involved in the process; thus nociceptive signals are generated by different structures.

Biostimulation parameters were administered on the medial field because predominantly the medial compartment of the joint is usually affected. This is predetermined by anatomical features, axis load, Q-angle, and rotation of the medial condyle of the knee in the last degrees of the range of motion.

Pain was measured by standard visual analogue scale. Dolorimetry was measured with standard Fisher dolorimeter.

Pedobarometric assessment was done for static and dynamic analysis of gait in the test and placebo group by RS footscan System, Belgium, with an active sensor area of 975 × 325 mm and 16 384 sensors and length of the active surface 2 m.

Static measurement was performed while patients stay motionless on the platform. The information that was obtained was the maximum pressure under the heel measured in N/cm^2^. The dynamic gait analysis was performed to assess the contact surface area:

- total plantar surface of each leg in cm^2^;
- the maximum pressure exerted under the heel of both feet in N/cm^2^.

These measurements are calculated directly on the software program. Then an analysis of the dynamics of the differences between the two legs was performed.

### 2.2. Statistical Analysis

All analyses were performed using Statistical Package SSPS (17 version). Methods of descriptive statistics, parametric, and nonparametric methods were used.

### 2.3. Characteristics of the Contingent

This study included 72 patients, divided into two groups, a test group (35 patients) and control group (37 patients). The average age of the patients from the control group is −64,71 ± 1,98 (mean ± Sd), 11 men (31,4%) and 24 women (66.6%).

## 3. Results

**Figure 1:**
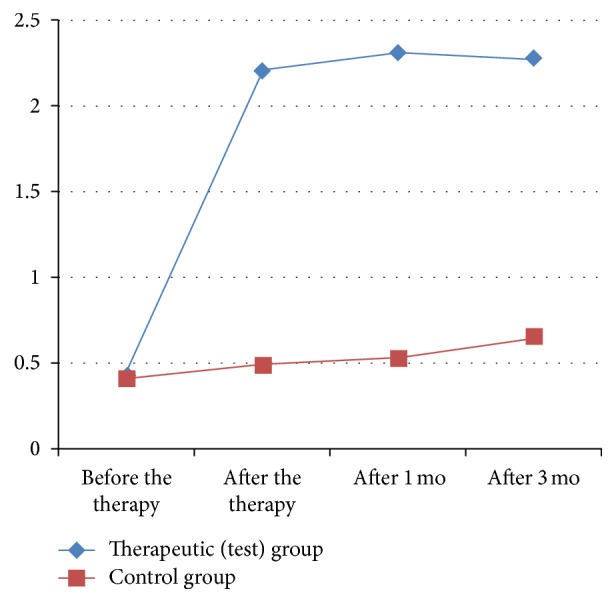
Dynamics of pain at rest in the test and control group [VAS].

**Figure 2:**
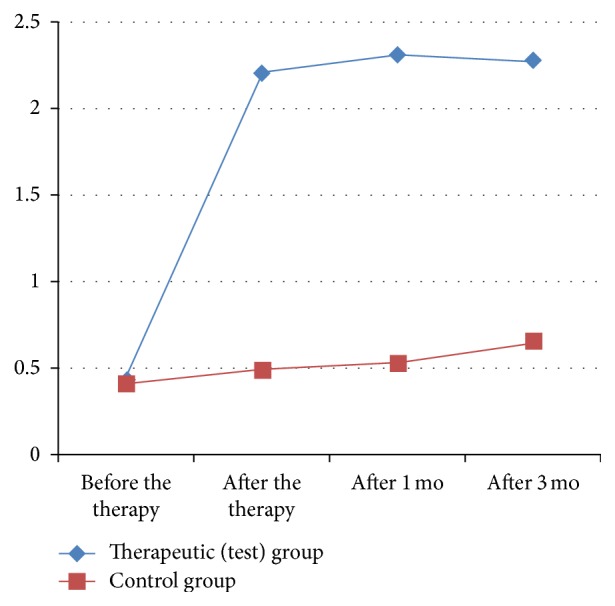
Dynamics of pain on palpation in the test and control group [VAS].

**Figure 3:**
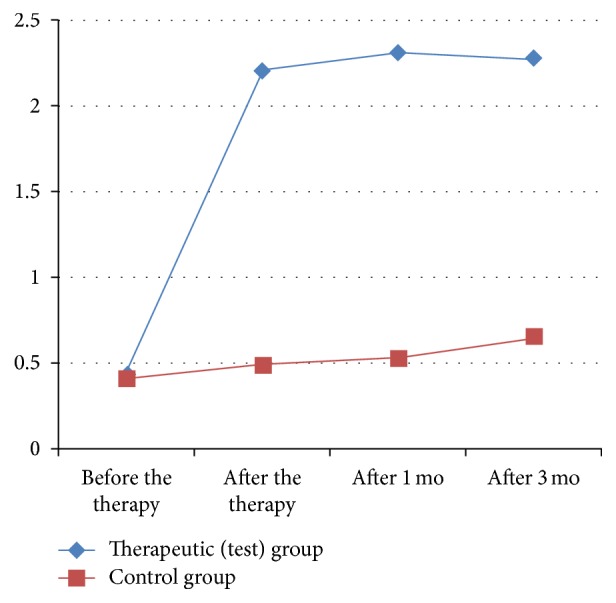
Dynamics of pain during movement in the test and control group [VAS].

**Figure 4:**
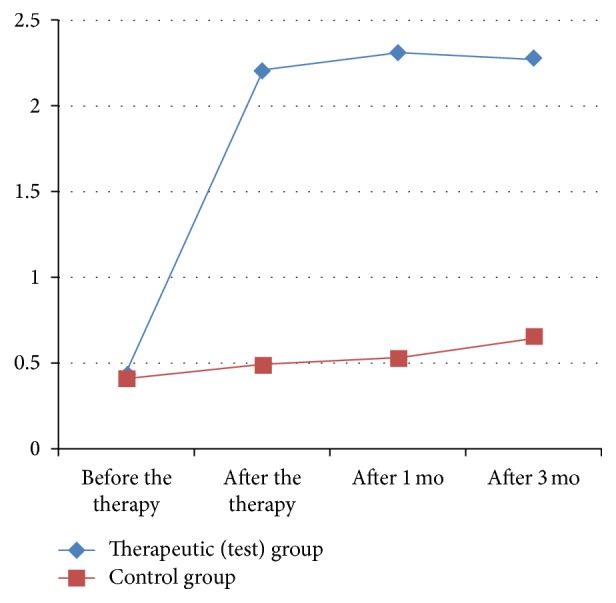
Dynamics of pain measured by dolorimetry in the test and control group [kg].

**Figure 5:**
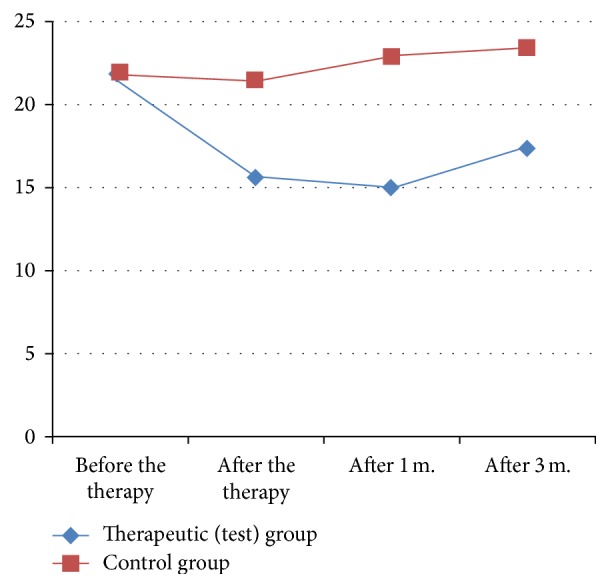
Pressure [N/cm^2^] static analysis.

**Figure 6:**
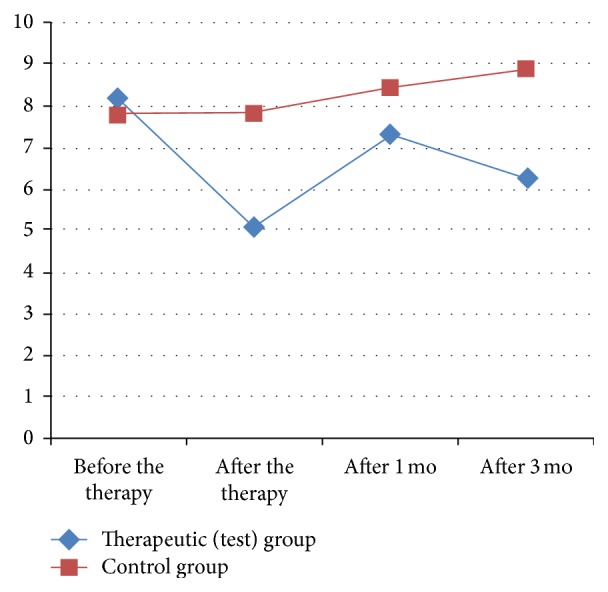
Contact surface area [cm^2^] dynamic analysis.

At baseline the groups were homogeneous, without statistically significant differences regarding age, severity of the OA, pain, and baropodometric assessments [Tables 1, 2, 3, 4, 5, and 6; Figures 1, 2, 3, 4, 5, and 6].

After the end of the therapy there was statistically significant reduction of pain at rest, pain on palpation, pain during movement, and pain, measured by dolorimetry in comparison with the baseline in both groups. But the dynamics of pain (reduction of pain in percentage) was significantly greater in the patients of the test group in comparison with the patients from the control group. The statistically significant difference regarding pain reduction between the groups was preserved at the follow-up one and three months later [Tables 1, 2, 3, and 4; Figures 1, 2, 3, and 4].

The data from the static pedobarometric assessment demonstrated that the difference in the pressure under the heel, comparing the affected and unaffected leg, is decreased significantly only in the patients from the test group.

The dynamic pedobarometric assessment demonstrated decrease of the difference in the contact surface area between the affected and unaffected leg also only in the test group. This gives us the confidence to conclude that the balance between the two legs in static position and during walking recovered only in the patients with high intensity laser therapy, thus leading to better functional improvement [Tables 5 and 6; Figures 5 and 6].

The analysis of the results in the test group found that high intensity laser therapy is effective for reducing pain in patients with osteoarthritis of the knee. There was an immediate effect after each procedure, which was demonstrated with clinical improvement of patients. There is also a cumulative effect after the 7-day course of treatment. The effect of laser therapy regarding pain reduction lasted for 3 months.

## 4. Discussion

The results demonstrated strong immediate, cumulative, and long lasting (for three months) effect of high intensity laser therapy on pain in knee osteoarthritis, which gives indication that high intensity laser therapy could be a promising new possibility in the treatment of osteoarthritis of knee.

The understanding of the pathogenic mechanisms and pathoanatomic changes in osteoarthritis raises the need of new therapeutic interventions in the process. The degeneration of the cartilage was found to be a result of intensive mechanical stress and lysis with the participation of mediators such as metalloproteinases, synthesized by chondrocytes: interleukin-1, prostaglandin E2, and proteinases 1, 3, and 13 [1, 2]. Тhe predominance of degradation process is a prerequisite for the development of osteoarthritis. In vitro studies found that this process could be modified by the application of growth factors of chondrocyte cultures, but these findings have not been proved in in vivo studies [2, 3, 8]. In OA there is functional inadequacy of the chondrocytes to synthesize the main components of the extracellular matrix and the collagen fibrils with quality, necessary to fulfil its primary biological role, hydrophility, elasticity, and compressivity of cartilage hyaline. The pathological process involves not only the cartilage and the underlying bone and synovial tissue, but also all the intra-articular and periarticular structures [1, 2, 8, 20].

Looking at the biological action of laser radiation at different diseases of the musculoskeletal system, the researchers defined as biological effect all the structural, biochemical, and functional changes occurring in a living organism after laser irradiation [10–13].

The interaction of bioobjects with laser radiation is determined by the characteristics of the radiation, wavelength, radiation mode (continuous or pulse), pulse duration, energy, and power. It was found that laser radiation in spectral range 600–1064 nm has the deepest penetration in tissues. For example, Nd-YAG laser penetration depth is up to 100 mm. The structure of the tissue changes the physical properties of laser radiation (coherency and polarization parameters). The properties of the biological object are specific: reflection and absorption coefficient, thermal conductivity and heat capacity, and presence of certain chemical compounds [2, 10, 11, 13].

The biological effect of laser radiation is associated with the following major effects: thermal (predominantly increases the temperature of the liquids, which leads to changes in the phase condition and intracellular pressure); mechanical (result of mechanical changes, kinetic and ultrasonic); electrical (induces changes in the structure of the molecules in the membrane and changes its permeability); photochemical (stimulation of photochemical reactions and selective absorption of the laser radiation of some chemicals in the cell); biostimulating (laser radiation supplies quantum energy to the cell without histological changes, i.e., without disruptive action). In this case, the cell uses the resulting energy for its own metabolism [10–13].

High intensity lasers have also thermal and mechanical effect and induce electromagnetic field, photoelectric, electrochemical, and other changes in the exposed tissues [12, 13].

Advantage of high intensity laser radiation in comparison with low intensity laser radiation is that with increasing the power the depth of penetration is increased, thus the effects in the deep structures, despite the retrogression of quantity and quality (coherence, polarization) of light electromagnetic energy [12, 13].

Pain relieving effect is realized by “Gate Control System” and a result of the stimulating effect of irradiation on regeneration of nerve fibers [12, 13].

The anti-inflammatory effect is realized by modulating the components of the inflammatory reaction, exudation, alteration, and proliferation, and also by stimulating the readaptive reactions of the organism. It is realized by blocking cyclooxigenases and lipooxigenases and impact on prostaglandin and prostacyclin synthesis. Cellular biostimulation is realized through accelerated cellular metabolism by increasing the mitotic index of the cells, which activate the reparative process. Extracellular ion transport is boosted by activating cell exchange. All of these mechanisms lead to beneficial effects regarding edema and stimulation of the healing process, trophycs, and venous and lymphatic microcirculation [8, 13].

The effectiveness of HILT is based on the specific and characteristic high peak power of the laser pulse with a certain frequency and pulse width. Thanks to this high peak power a large amount of energy is supplied for a short time (vertical effect), in contrast to the traditional delivery of the same amount of energy for a long time and the risk of heating and tissue damage (horizontal effect) [10, 11].

Studies contributing to the understanding of molecular mechanisms and cellular processes underlying systemic effects produced by pulsed Nd: YAG laser irradiation lead to an understanding of the factors, but so far the results are not definitive. Consequently, due to the lack of effective chromophore absorption of Nd: YAG radiation (wavelength 1064 nm) in cells and tissues, it is assumed that, instead of photochemical processes, mechanisms of absorption are probably due to the combined photomechanical and thermodynamic interactions responsible for analgesic, antioedematous, anti-inflammatory, and reparative effects of pulsed Nd: YAG laser. It is assumed that the cells “responsive” to the pulsed Nd: YAG laser irradiation react to it by mechanotransductive mechanism and interaction between the tissue and the laser radiation alters the mechanics of the microenvironment of the cell, thereby acting on the cells by mechanical stress. Biological effects of high-energy lasers include mechanical shock effect, thermal effect, occurrence of electromagnetic field, and photovoltaic, electrochemical, and other changes in exposed tissues [10, 11, 13].

A lot of studies were conducted with cell cultures to track the ultrastructural changes and the effect of therapeutic nondestructive parameters of high-intensity laser radiation on tissue.

To our knowledge this is one of the first placebo controlled studies comparing the effect of HILT with placebo application in patients with osteoarthritis of the knee. The method of application of HILT that we introduced is refined according to the specific pathology. Pedobarometric analysis was used for objective assessment of the dynamics in statics and locomotion as a result of the treatment.

*Limitations of the Study*. The number of patients and the lack of methods for assessment of the structural changes in the knee joint as a result of treatment with HILT could highlight the exact mechanism of its effect in the treatment of osteoarthritis.

## 5. Conclusion

Based on the results of our pilot study we may conclude that high intensity laser therapy could be recommended as a treatment of choice for reduction of pain and improving of function in patients with osteoarthritis of the knee. Further studies are needed to clarify the best treatment protocol and the long-term results.

## Figures and Tables

**Table 1:** Comparison of the dynamics of pain at rest at baseline, after the end of the treatment, and during the follow-up one and three months later [*P* < 0.0001].

Indicators	Groups	Before treatment *t*_0_ Mean ± SE	*P* value	Dynamics at the end of treatment Δ_*t*_1_−*t*_0__ (%)	*P* value	Dynamics 1 month after treatment Δ_*t*_2_−*t*_0__ (%)	*P* value	Dynamics 3 months after treatment Δ_*t*_3_−*t*_0__ (%)	*P* value
Pain at rest [VAS]	Test	3.51 ± 0.14	*N*	−83.87	<0.0001	−86.38	<0.0001	−80.95	<0.0001
	Control	3.50 ± 0.25		−25.42		−36.42		−11.37	

**Table 2:** Comparison of the dynamics of pain on palpation at baseline, after the end of the treatment, and during the follow-up one and three months later [*P* < 0.0001].

Indicators	Groups	Before treatment *t*_0_ mean ± SE	*P* value	Dynamics at the end of treatment Δ_*t*_1_−*t*_0__ (%)	*P* value	Dynamics 1 month after treatment Δ_*t*_2_−*t*_0__ (%)	*P* value	Dynamics 3 months after treatment Δ_*t*_3_−*t*_0__ (%)	*P* value
Pain on palpation [VAS]	Test	6.13 ± 0.12	N.S	−62.94	<0.0001	−71.76	<0.0001	−74.92	<0.0001
	Control	6.28 ± 0.10		−2.79		−8.92		−3.83	

**Table 3:** Comparison of the dynamics of pain during movement at baseline, after the end of the treatment, and during the follow-up one and three months later [*P* < 0.0001].

Indicators	Groups	Before treatment *t*_0_ mean ± SE	*P* value	Dynamics at the end of treatment Δ_*t*_1_−*t*_0__ (%)	*P* value	Dynamics 1 month after treatment Δ_*t*_2_−*t*_0__ (%)	*P* value	Dynamics 3 months after treatment Δ_*t*_3_−*t*_0__ (%)	*P* value
Pain on movement [VAS]	Test	8.47 ± 0.23	N.S	−67.82	<0.0001	−72.16	<0.0001	−68.51	<0.0001
	Control	8.42 ± 0.19		−8.52		−20.35		−10.82	

**Table 4:** Comparison of the dynamics of pain measured by dolorimetry at baseline, after the end of the treatment, and during the follow-up one and three months later [*P* < 0.0001].

Indicators	Groups	Before treatment *t*_0_ mean ± SE	*P* value	Dynamics at the end of treatment Δ_*t*_1_−*t*_0__ (%)	*P* value	Dynamics 1 month after treatment Δ_*t*_2_−*t*_0__ (%)	*P* value	Dynamics 3 months after treatment Δ_*t*_3_−*t*_0__ (%)	*P* value
Pain measured with dolorimeter (kg)	Test	0.44 ± 0.03	N.S	6.14	<0.0001	6.48	<0.0001	6.73	<0.0001
	Control	0.41 ± 0.04		1.35		1.49		1.45	

**Table 5:** Dynamics of the difference in the static pressure of the feet at baseline, after the end of treatment, and one month and three months later in the test and control groups.

Indicators	Groups	Before treatment *t*_0_ mean ± SE	*P* value	Dynamics at the end of treatment Δ_*t*_1_−*t*_0__ (%)	*P* value	Dynamics 1 month after treatment Δ_*t*_2_−*t*_0__ (%)	*P* value	Dynamics 3 months after treatment Δ_*t*_3_−*t*_0__ (%)	*P* value
Pressure – static [N/cm^2^]	Test	21.29 ± 1.35	N.S	−28.45	<0.0001	−23.38	0.016	−19.94	<0.0001
	Control	21.88 ± 1.03		−1.63		5.34		7.79	

**Table 6:** Dynamics of the difference in the contact surface are at baseline, after the end of treatment, and one month and three months later in the test and control groups.

Indicators	Groups	Before treatment *t*_0_ mean ± SE	*P* value	Dynamics at the end of treatment Δ_*t*_1_−*t*_0__ (%)	*P* value	Dynamics 1 month after treatment Δ_*t*_2_−*t*_0__ (%)	*P* value	Dynamics 3 months after treatment Δ_*t*_3_−*t*_0__ (%)	*P* value
Surface area [cm^2^]	Test	8.17 ± 0.40	N.S	−37.04	<0.0001	−15.95	<0.0001	−22.44	<0.0001
	Control	7.81 ± 0.03		1.07		9.61		16.32	
